# A case report of CT-diagnosed renal infarct secondary to syphilitic aortitis

**DOI:** 10.1186/s12879-017-2624-1

**Published:** 2017-07-26

**Authors:** Maaike Spaltenstein, Françoise Humbert, Diem-Lan Vu, Ilker Uçkay, Gregor John

**Affiliations:** 1 Division of Internal Medicine, Hôpital Neuchâtelois, La Chaux-de-Fonds, Switzerland; 2 Division of General Internal Medicine, University Hospitals of Geneva, Geneva, Switzerland; 3 Division of Infectious Diseases, University Hospitals of Geneva and Faculty of Medicine, University of Geneva, Geneva, Switzerland

**Keywords:** Aortitis, Syphilis, Renal infarct, Thrombus, Pet, Tertiary

## Abstract

**Background:**

Even though reported cases of syphilis have been increasing, cases of tertiary syphilis remain extremely rare. The majority of our knowledge with regard to complications of syphilis such as aortitis was acquired before the advent of relatively modern technologies such as CT, MRI and PET. This case report presents a rare case of syphilitic aortitis associated with a renal infarct caused by a peripheral arterial embolism diagnosed by CT.

**Case presentation:**

We present a young man with sudden abdominal pain and flank tenderness without fever. Blood tests showed acute kidney failure. Computed tomography showed a right renal infarct and a non-circular thickening of the descending thoracic aortic wall with intra-luminal thrombus. Serology confirmed the diagnosis of syphilis. Treatment with anticoagulant and penicillin resulted in a good outcome. Follow-up PET-MRI showed resolution of the thrombus with a metabolically inactive atheromatous plaque.

**Conclusion:**

Technologies, such as CT, PET-CT and PET-MRI, that were not present during the pre-antibiotic era, can provide new insights into rare presentations of tertiary syphilis such as aortitis. These imaging modalities show promise for early radiological diagnosis of aortitis in syphilis and may be useful for determining the response to treatment in specific cases.

**Electronic supplementary material:**

The online version of this article (doi:10.1186/s12879-017-2624-1) contains supplementary material, which is available to authorized users.

## Background

After having been on the verge of eradication in 2000, with the lowest-ever rate of 2.1 per 100,000 population per year, [1] the worldwide incidence of syphilis has been increasing, culminating in the current rate of 5.1 cases per 100,000 population per year in Europe [2]. This trend is mainly due to an increased number of cases among men who have sex with men, and to changes in sexual behaviour [2].

Known as the “great imitator” with a multitude of rare presentations, syphilis concerns almost all medical disciplines and should be included in the differential diagnosis of bizarre cases.

The spirochete *Treponema pallidum* is the agent of syphilis, and is probably limited to a human reservoir. Transmission predominantly occurs through sexual intercourse, although other transmission routes such as vertical transmission or mucous contact with the infectious chancre have been described.

Clinical presentation depends on the infection stage. Primary syphilis, with the indolent chancre, usually appears 21 days (10 to 90 days) after exposure and may spontaneously resolve after 1 to 4 months. Secondary syphilis corresponds to the dissemination of treponemal bacteria and occurs 3 to 6 months after the chancre with a macular rash on the trunk, face, palms and soles. Other manifestations include fever, headache, malaise, anorexia, diffuse lymphnodes, joint inflammation, hepatitis, uveitis, and hair loss. When left untreated, 30% of cases evolve within roughly 10 to 40 years to the tertiary stage, and manifest as infections of the central nervous system (neurosyphilis), skin and subcutaneous tissue (gummas), or as cardiovascular infections. While only 10 to 15% of patients will develop clinical signs, [3] cardiovascular involvement is the main cause of death attributable to syphilis [3, 4]. Although efforts to devise diagnostic tools for the early detection of cardiovascular involvement have been sought after with modest success using conventional radiography [5], post-mortem autopsies have historically been the only approach available to confirm cardiovascular syphilis [5–7]. Early studies, including the Tuskegee Study on African-American men showed evidence of aortitis in about half of autopsied subjects [6]. However, in the sixties only 17% of syphilitic aortitis were diagnosed before necropsies [7]. Since the advent of antimicrobial therapies, the focus has shifted from finding specific lesions caused by the syphilis to treatment with resolution of all lesions, specifically identified or not. As a consequence, the expected radiological findings have not been subject to large-scale studies, and the early diagnosis of syphilitic aortitis using modern radiological equipment has not been fully explored.

## Case description

**Fig. 1:**
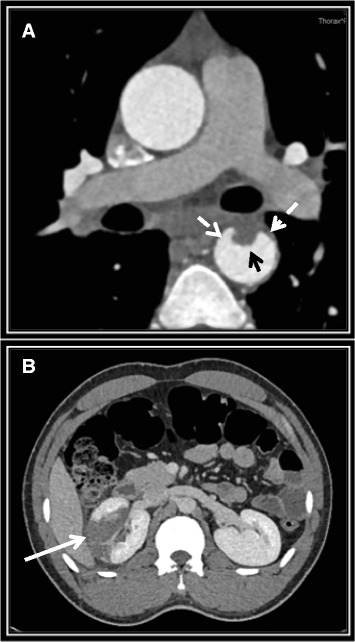
Computed tomography angiography of the chest and abdomen. It showed a non-circular thickening of the aortic wall of the descending thoracic aorta (dashed arrows) associated to an intra-luminal thrombus (*black solid arrow*) (**a)**, and a right renal infarct (solid arrow) (**b**)

A 27-year-old man from Mali, known for untreated chronic hepatitis B, was admitted to the emergency department for sudden abdominal pain and flank tenderness without fever (see Additional file 1: Figure S1). The rest of the physical examination was normal, including the genital region. Blood tests revealed a high leucocyte count (15 G/L), an elevated serum C-reactive-protein level (179 mg/l) and acute kidney failure (creatininemia 125 μmol/l). Computed tomography showed a right renal infarct (Fig. 1) and a non-circular thickening of the descending thoracic aortic wall with intra-luminal thrombus (Fig. 1). The patient was heterosexual and denied any risky sexual behaviour. He remembered having had a painless ulceration of the penis some time ago without further precision. The aortic lesion in an otherwise healthy young man led us to consider syphilis. Serology confirmed the diagnosis of syphilis with a *Treponema* antibody index of 14.3 (Normal <0.80), rapid plasma reagin of 4 (RPR or VDRL, Normal <1 titer), and a *Treponema pallidum* hemaglutination assay titer of 2560 (TPHA, Normal <80 titer). HIV testing and TB IGRA were negative, as were investigations for autoimmune and thrombophilic disorders, and cardiovascular disease. Except for moderate smoking, the patient didn’t have any cardiovascular risk factors.

We concluded that a thrombotic embolism originating from the syphilitic aortitis caused the renal infarct. Therapeutic anticoagulation and intravenous penicillin G (4 million units every 4 h) during 2 weeks resulted in clinical resolution. Two weeks later, a PET-MRI scan showed complete resolution of the thrombus and a thin metabolically inactive atheromatous plaque. Other causes of aortitis such as rheumatologic diseases or other infective aortitis seemed less likely given the absence of systemic symptoms, negative antibody work-up, positive syphilis serology and the favorable outcome following treatment by penicillin. Over the course of a two-year follow-up RPR titer progressively became negative, while TPHA titers decreased to 1280 within 4 months and remained stable until the end of the follow-up.

## Discussion

Syphilitic aortitis is the cardinal lesion of syphilitic cardiovascular disease. Usually asymptomatic, syphilitic aortitis can manifest as an aneurysm, aortic insufficiency, coronary stenosis or mural thrombosis. Potential complications are rupture, left ventricular hyperthrophy, or myocardial infarct [3, 8]. Unlike our case, aortitis more frequently affects the ascending aorta. Embolic events due to an aortic thrombus are scarce, but have been published [9, 10]. In addition, although the median time between infection and aortitis is 20 years, in a cohort of syphilitic patients more than 14% had aortitis within 3 years after the appearance of the chancre [5].

Diagnosis of syphilitic aortitis is classically based on serology, conventional radiography or autopsies [3, 8]. The majority of our knowledge with regard to complications of syphilis was acquired before the advent of relatively modern technologies such as computed tomography. The diagnosis should differentiate between major causes of aortitis, mainly other infective aortitis, large vessel vasculitis (Takayasu arteritis or Giant Cell Arteritis), and aortitis less frequently associated with other rheumatologic diseases (e.g.: systemic lupus erythematous, rheumatoid arthritis, ANCA-associated vasculitis). With the great decrease of syphilis cases in the post antibiotic era, the radiological findings present on modern imaging studies such as CT, PET CT and PET MRI are not fully explored. These technologies can give a new insight into rare presentations of syphilis such as aortitis and help in differentiating it from alternate aetiologies, especially when the location is infrequent (e.g.: descending aorta) [11]. These technologies have already demonstrated utility in aortitis with a rheumatologic origin such as Takayasu arteritis and Giant Cell Arteritis where FDG PET is a reliable marker for inflammation while CT and MRI provide more precise anatomic localisation [12]. MRI has better resolution of the vessel wall and oedema thereof, and unlike CT, does not expose to radiation or iodinated contrast media [12]. Modern imaging techniques might also, for example, allow early radiological diagnosis, and be useful for determining the response to treatment of syphilitic aortitis. A decrease in 18F–FDG uptake in repeat PET-CT scans has been reported at 24 weeks post diagnosis, and at the end of antibiotic therapy, which usually lasts 3 weeks [13, 14]. Nevertheless, the sensitivity, specificity, and usefulness of nuclear imaging for the diagnosis and follow-up of syphilitic aortitis needs to be further investigated.

## Conclusion

This case report presents a rare case of syphilitic aortitis associated with a renal infarct caused by a peripheral arterial embolism. Given the proteiform presentation of syphilis, physicians must consider this diagnosis when faced with atypical signs, symptoms and even radiological findings. New imaging techniques such as 18F–FDG PET-CT or PET-MRI show promise for early radiological diagnosis of aortitis in syphilis and possibly treatment response, even in patients initially considered as showing no signs or symptoms of syphilis. In addition, physicians interpreting such newer imaging modalities must consider syphilis in their differential diagnosis of aortitis.

## Additional file

Additional file 1: Timeline. (DOCX 37 kb)

## Abbreviations

18F–FDG PET-CT: F-18 fluoro-2-D-deoxyglucose PET scans with computed tomography
CT: Computed tomography
HIV: Human immunodeficiency virus
MRI: Magnetic resonance imaging
PET: Positron emission tomography
RPR: Rapid plasma reagin
TB IGRA: Interferon gamma release assays for tuberculosis
TPHA: Treponema pallidum hemaglutination assay
VDRL: Venereal disease research laboratory
