# Supplementary material for: A case report of CT-diagnosed renal infarct secondary to syphilitic aortitis
Source: BMC Infect Dis. 2017 Jul 26;17:520. doi: 10.1186/s12879-017-2624-1 (PMC5530486; doi:10.1186/s12879-017-2624-1)
Supplement: Additional file 1: — Timeline. (DOCX 37 kb) [file 12879_2017_2624_MOESM1_ESM.docx]

November 23, 2013

November 23, 2013

November 23, 2013

No symptoms

Negative rapid plasma reagin (RPR or VDRL)

PET-MRI scan: complete resolution of the thrombus and a thin metabolically inactive atheromatous plaque

Clinical resolution

Therapeutic anticoagulation and intravenous penicillin G (4 million units every 4h) during two weeks

November 27, 2013

Positive syphilis serologies

Right side flank tenderness Inflammatory syndrome

Acute kidney failure

CT : right renal infarct

non-circular thickening of the descending thoracic aortic wall with intra-luminal thrombus

Follow-up of 4 years, the patient was not admitted to the hospital

27-year-old man from Mali, known for untreated chronic hepatitis B

February 1, 2016

December 12, 2013

December 12, 2013

November 27, 2013

Emergency room consultation for sudden abdominal pain without fever
